# Vertebral artery hypoplasia and hemodynamic impairment in transient global amnesia: a case control study

**DOI:** 10.3389/fneur.2024.1398352

**Published:** 2024-05-09

**Authors:** Ralph Werner, Alexandra Ekstrom, Ingo Kureck, Johannes C. Wöhrle

**Affiliations:** ^1^Neurologie und Stroke Unit, Katholisches Klinikum Koblenz-Montabaur, Koblenz, Germany; ^2^Neurologie, Klinische Neurophysiologie und Stroke Unit, Unfallklinik Murnau, Murnau am Staffelsee, Germany; ^3^Klinik für Diagnostische und Interventionelle Radiologie/Nuklearmedizin, Katholisches Klinikum Koblenz-Montabaur, Koblenz, Germany; ^4^Radiologisches Institut Dr. von Essen, Koblenz, Germany; ^5^Neurologische Klinik, Medizinische Fakultät Mannheim, Universität Heidelberg, Mannheim, Germany

**Keywords:** transient global amnesia, vertebral artery hypoplasia, magnetic resonance imaging angiography, hemodynamics, hippocampal perfusion

## Abstract

**Introduction:**

The aetiology of transient global amnesia (TGA) is still a matter of debate. Besides ischemia of the mesial temporal lobe including the hippocampus, migraine-like mechanisms, epileptic seizures affecting mnestic structures, or venous congestion in the (para) hippocampal area due to jugular vein insufficiency have been discussed. We assessed the diameters of the intracranial arteries of TGA patients compared to controls to identify differences that support the hypothesis of reduced hippocampal perfusion as a pivotal factor in the pathophysiology of TGA.

**Methods:**

We reviewed magnetic resonance imaging time of flight angiographies (TOF-MRA) that were acquired during in-patient treatment of 206 patients with acute TGA.

**Results:**

The diameters of the vertebral artery (VA) in the V4 segment, the proximal basilar artery, and the internal carotid arteries were measured manually. We compared the findings with TOF-MRA images of an age and sex matched control group of neurological patients without known cerebrovascular pathology. In TGA patients the diameter of the right VA was significantly (*p* < 0.01) smaller compared to controls (2.09 mm vs. 2.35 mm). There were no significant differences in the diameters of the other vessels. Only the fetal variant of the posterior cerebral artery was slightly more common in TGA.

**Discussion:**

The smaller diameter (hypoplasia) of the right VA supports the hypothesis of a contribution of hemodynamic factors to the pathophysiology of TGA. The fact that hypoplasia represents a congenital condition might be the explanation why previous studies failed to find an increased rate of the classical (acquired) vascular risk factors.

## Introduction

Transient global amnesia (TGA) is a neurological disorder, usually self-limiting within 24 h, featuring acute anterograde amnesia as the most striking clinical symptom. For patients, the experience of TGA is at least strange, for people next to them it may appear alarming, and for the treating neurologists it is a fascinating syndrome.

The etiology of TGA is still unclear. Focal ischemia of the hippocampus and related structures, an association with migraine or migraine-like functional dysregulation, and epileptic seizures affecting the mnestic structures have been proposed (1–6). As TGA is often preceded by physical exertion (sports activities, heavy work, cold shower, sexual intercourse) or mental stress (e. g., acute excitement from anger or grief) that both may lead to an increase of intrathoracic pressure, secondary venous congestion in the mesial temporal lobe has also been suggested, facilitated by a Valsalva maneuver or jugular vein insufficiency in certain circumstances (7–11).

Several imaging studies demonstrated that uni- or bilateral small punctate hippocampal lesions can be detected with high-resolution diffusion weighted (DWI) magnetic resonance imaging (MRI) in about 2/3 of TGA patients (12, 13).

A circadian rhythm (with a peak of attacks in the morning) but not a seasonal clustering has been described (14).

While several guidelines allow a diagnosis based on clinical evaluation only, in about 10% of patients presenting with the clinical picture of TGA either an alternative diagnosis or a severe comorbidity with a probable impact on the occurrence of the amnestic episode can be found by thorough diagnostic investigations (15–17).

Inspired by single-case observations of hypoplastic vertebral arteries in patients with TGA, we designed the present study in which we assessed the diameters and the frequency of hypoplasia of the large cerebral arteries supplying the hippocampus of TGA patients (compared to controls) as surrogates for a potentially reduced hippocampal perfusion as a pivotal contributor to the pathophysiological cascade of TGA.

## Materials and methods

We evaluated magnetic resonance imaging (MRI) time of flight angiographies (TOF-MRA) of consecutive patients with TGA that were acquired during in-patient treatment in a German academic teaching hospital between January 2008 and December 2017. The patients were identified by a retrospective search of our hospital information system (ORBIS, Agfa HealthCare, Germany) for in-patients with the main diagnosis “transient global amnesia” (ICD-10 G45.4).

We compared the results of the TGA patients with TOF-MRA images of an age and sex matched control group of neurological patients. Patients as well as controls with known cerebrovascular pathologies (e. g., stenosis of the examined arteries, acute or recent stroke, assessed by medical history, duplex-sonography and CT-angiography) were excluded from the analysis.

All MRI examinations were performed on a 1.5 T MR scanner (Signa HDxt, GE Healthcare, United States) with a standard stroke protocol including TOF-MRA (slice thickness 1.6 mm) of the intracranial arteries.

**Figure 1:**
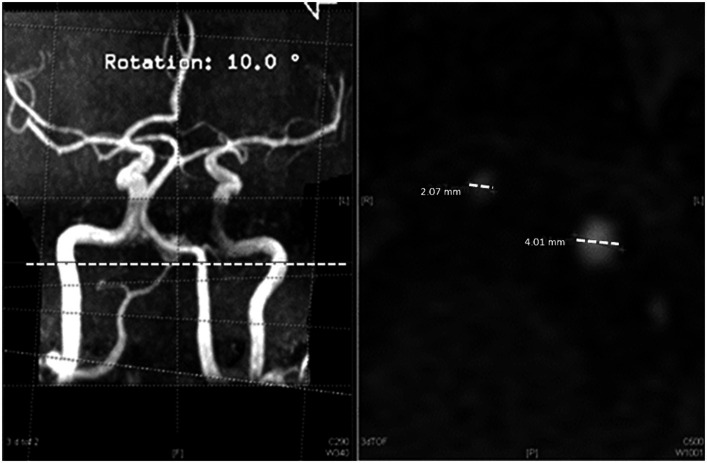
Diameter assessment with the centricity enterprise web 3.0 viewer.

The vessel diameters were measured by three experienced neurologists (RW, AE, JW) and one neuroradiologist (IK), respectively on interpolated axial 2D source images according to a standardized protocol: The V4 segment of the vertebral arteries (VA) and the proximal basilar artery (BA) were analyzed 0–10 mm proximal/distal to the vertebrobasilar junction. The intracranial internal carotid arteries (ICA) were evaluated on the same level as the VA. The reviewers were free to select the image which displayed the best cross-section for measurement within the above-mentioned range. If the cross-sections did not have a round but an ovoid or longitudinal shape, the diameter of the shorter axis through the visually estimated midpoint of the vessel was chosen. The measurements were performed manually using the Centricity Enterprise Web 3.0 viewer (GE Healthcare, United States) with maximum zoom factor (Figure 1).

The posterior cerebral artery (PCA) was classified as fetal type (fPCA) when its major stem arose from the ipsilateral internal carotid artery and the diameter of the P1 segment of the PCA was smaller than the diameter of the posterior communicating artery (PcomA), respectively.

The reviewers were blinded for the status (TGA or control) of the imaging dataset.

For the statistical analysis we calculated the mean diameters for the TGA and the control group and the corresponding standard deviations. For the group comparisons of continuous variables meeting the assumptions of normality we used a two-sided *t*-test for unpaired samples. For the dichotomous variables, odds ratios were calculated. A *p*-value <0.05 was defined as the threshold of statistical significance.

As the retrospective analysis of the anonymized data (that had been acquired exclusively according to our routine treatment protocol) was in accordance with the directive of the local ethics commission (Rhineland-Palatinate Medical Council) no separate approval was required.

## Results

In total, we identified 206 patients in the ten-year period with the diagnosis of acute TGA who had an MRI scan during in-patient treatment. The average age was 67 years, 119 were women (58%), 87 were men (42%).

In TGA patients, the diameter of the right VA was significantly (*p* < 0.01) smaller compared to controls (2.09 mm vs. 2.35 mm). There were no significant differences in the diameters of the other vessels.

We also calculated the ratios of the diameters of both VA as an indicator of asymmetry which was significantly higher in the TGA group (1.18 vs. 1.02, *p* < 0.05). The ratio of the diameters between the sum of the posterior circulation vessels (V4 R + V4 L + BA) and the sum of the anterior circulation (ICA R + ICA L) was not different in both groups (Table 1).

**Table 1:** Baseline data and vessel diameters.

	TGA	Controls	*p*-value
*n*	206	206	
Sex			
Female, *n* (%)	119 (58)	119 (58)	NA
Male, *n* (%)	87 (42)	87 (42)	NA
Age in years			
Mean (SD)	66.8 (8.9)	66.7 (9.2)	0.835
Min-max	38.5–86.7	39.6–86.4	NA
Diameters in mm (SD)			
ICA R	4.58 (0.52)	4.48 (0.55)	0.066
ICA L	4.48 (0.54)	4.45 (0.55)	0.554
V4 R	2.09 (0.96)	2.35 (0.84)	0.004^*^
V4 L	2.52 (0.93)	2.43 (0.86)	0.309
BA	3.28 (0.68)	3.26 (0.66)	0.731

^*^Significant difference including Bonferroni correction for multiple comparisons; NA, not applicable; °aplastic vessels excluded from calculation; R, right; L, left.

Using a cut-off diameter of 2.5 mm for the definition of VAH, we found no significant difference of the proportions of VAH between TGA patients and controls. When calculating with a lower value of 2.0 mm (which was closer to the medium diameter of the right V4 in our TGA cohort), VAH was significantly more frequent in the TGA group. This difference was even more evident, when only the absence of V4 (aplasia) was considered (Table 2).

**Table 2:** Prevalence of VAH in TGA and controls.

	TGA	Controls	OR (95% CI)
V4 ≤ 2.5 mm, *n* (%)			
R	133 (65)	119 (58)	1.33 (0.90–1.98)
L	83 (40)	101 (49)	0.70 (0.48–1.04)
R or L	158 (77)	159 (77)	0.97 (0.62–1.54)
V4 ≤ 2.0 mm, *n* (%)			
R	84 (41)	59 (29)	1.72 (1.14–2.59)^*^
L	41 (20)	51 (25)	0.76 (0.47–1.20)
R or L	109 (53)	95 (46)	1.31 (0.89–1.93)
V4 aplasia, *n* (%)			
V4 R	20 (10)	9 (4)	2.35 (1.05–5.30)^*^
V4 L	14 (7)	11 (5)	1.29 (0.57–2.90)
R or L	32 (16)	19 (9)	1.81 (0.99–3.31)

^*^Significant difference; R, right; L, left.

The prevalence of the fetal variant of the right posterior cerebral artery (fPCA) was significantly lower in the group of all TGA patients compared to our controls, but this relation was not observed in the subgroup of patients with a right VA with a diameter ≥ 2.0 mm (Table 3).

**Table 3:** Prevalence of fPCA in TGA and controls.

	TGA	Controls	OR (95% CI)
fPCA, n (%)			
R	32 (16)	49 (24)	0.59 (0.36–0.97)^*^
L	38 (18)	34 (17)	0.11 (0.69–1.90)
R + L	10 (5)	13 (6)	0.76 (0.32–1.77)
R or L	60 (29)	70 (34)	0.80 (0.72–1.11)
V4 R ≤ 2.0 mm, n (%)			
+fPCA R	16 (19)	16 (27)	0.63 (0.29–1.40)
+fPCA L	20 (24)	11 (19)	1.36 (0.69–3.11)
+fPCA R + L	8 (10)	5 (8)	1.09 (0.34–3.52)
+fPCA R or L	30 (36)	21 (36)	1.01 (0.50–2.02)

^*^Significant difference; R, right; L, left.

## Discussion

In our cohort of 206 consecutive patients with acute TGA the diameter of the right VA was significantly smaller compared to an age and sex matched control group of neurological patients. The diameters of the other vessels studied, the left VA, the BA, and both ICA did not show such a difference.

The average diameter of the right VA of 2.09 mm in our cohort fulfils the criterion of hypoplasia that is used in many studies, though there is no generally accepted standard definition of vertebral artery hypoplasia (VAH). Recent studies from Germany and Switzerland on VAH in the context of posterior circulation stroke using MRA used a cut-off value of 2.5 mm or a ratio of more than 1 to 1.7 compared to the contralateral vessel (18, 19).

VAH and asymmetry are common conditions in the general population and usually the left VA has the greater caliber. Peterson et al. (20) found that VAH can be reliably detected by MRI techniques and described a prevalence of 43.5%, out of which 80.7% were located on the right side. Other authors reported VAH prevalences between 14 and 62% (depending on the criteria used) with a moderately genetic determination in twin studies (21, 22). Kulyk et al. (23) found an increased VAH prevalence of 33.7% in 193 patients with posterior circulation stroke compared to 14.1% in patients with anterior circulation stroke; in their cohort, right sided hypoplasia was three times more frequent than left sided hypoplasia.

In our study the prevalence was even higher. When using a cut-off value of 2.0 mm we found VAH (either on the right or the left side) in 53% of the TGA patients (and in 46% of the controls). Right sided hypoplasia was 1.4 times more frequent than left sided hypoplasia.

However, the comparability of the various studies is limited due to the inconsistent definitions of VAH and the different ways of assessment, e. g. ultrasound, flow-based MR techniques (like TOF-MRA, as used in the present study), or anatomically based MR imaging (T1 with contrast agents).

The finding of only right sided VAH as a risk factor for TGA might be explained by the fact that right sided VAH is also more common in the normal population (like in our control group, respectively), suggesting that the right VA is in principle more susceptible for caliber variations than the left one. Higher shear stress during embryogenesis (because the left subclavian branches sprout directly from the aortic arch), leading to a dominance of the left VA, has been proposed as an explanation; an association with the higher blood demand of the dominant left cerebral hemisphere and the left sided vertebral artery dominance has not been proven (24).

A smaller VA diameter can serve as a surrogate parameter for reduced perfusion, as blood flow is strongly related to a vessel’s diameter and VAH is associated with a decrease in net vertebral flow volume (25, 26). Therefore, a smaller VA diameter might be regarded as a risk factor for reduced downstream perfusion, like in the hippocampal area within the posterior cerebral artery territory. Under resting state condition, the reduced flow volume of a hypoplastic VA can be compensated by merging with the flow volume of the contralateral VA, but a reduced autoregulatory ability of the hypoplastic artery might lead to (hippocampal) hypoperfusion in conditions of a higher demand of blood supply. Additionally, stress hormones like adrenaline and cortisol (that are released in TGA triggering events) lead to vasoconstriction which might have a more deleterious effect in a hypoplastic vessel due to threshold phenomena of perfusion.

Based on SPECT scans, performed shortly after onset of TGA in 6 patients, Kim et al. suggested 2016 “… that the underlying mechanism of TGA may be temporary ischemia in the hippocampus and thalamus” (27).

Perosa et al. used 7 T ultrahigh-field MRI to differentiate two forms of hippocampal vascular supply: A single supply by the posterior cerebral artery (PCA) and a mixed type with inflow from the anterior choroidal artery (AChA), a distal branch of the internal carotid artery, as well as from the PCA. They found “… a positive effect of having a mixed supply in at least one hippocampus” that resulted in better cognitive performance (compared to single hippocampal supply). Their conclusion was that a decrease in perfusion in single supplied hippocampi might rather lead to ischemia, especially in the anterior part of the hippocampus (28).

Gaigalaite et al. (29) reported that fPCA is associated with ipsilateral VAH (based on the analysis of MRA data of 923 healthy volunteers). In the present study, the prevalence of the right fPCA variant was slightly lower in the TGA group compared to controls. This finding seems plausible because fPCA could enhance hippocampal blood supply by permitting additional perfusion from the anterior circulation in case of a reduced vertebral perfusion. Thus, the presence of a right fPCA might represent a protective factor for TGA.

The fact that hypoplasia represents a congenital condition might be the explanation why previous studies neither found a higher rate of the classical, mostly acquired, vascular risk factors (high blood pressure, diabetes, hyperlipidaemia) nor an increased risk of stroke in patients with TGA compared to the normal population (which is often used as a strong argument against a vascular pathology) (30). However, this view has recently been challenged, when a large Korean propensity-matched cohort study (including more than 10.000 patients with TGA) found evidence for an increased risk of cerebral ischemia after TGA in a follow-up period of 10 years, a finding that supports our hypothesis (31).

As not all TGA patients have VAH, it is obvious that a smaller VA diameter represents only one risk factor among others, preferably in coincidence with other conditions. For example, VAH has been found to be also a risk factor in posterior circulation stroke or vestibular neuritis (23, 32). Nevertheless, it is not possible to calculate its role in individual cases. As much as on an even larger scale, arterial hypertension is undoubtedly a risk factor for cerebral ischemia, but not every hypertensive individual will suffer a stroke sooner or later.

Like other entities that have been proposed as risk factors for TGA (physical or psychological stress, migraine, jugular vein incompetence), VAH seems to be another contributor to a multifactorial pathophysiological cascade.

Based on our findings, we tried to create a model that integrates the two recently most debated risk factors: stress and jugular vein insufficiency (7, 9, 23, 33).

A hypothetic cascade leading to the clinical manifestation of TGA would start with an enhanced metabolic demand in the hippocampus due to stress from an external or internal trigger, while on the other hand increased levels of circulating glucocorticoids promote a decrease in the blood flow of the mesial temporal lobe (which has already been demonstrated by de Quervain et al. (34) by positron emission tomography in 2003). This regional hypoperfusion might be aggravated by a reduced arterial inflow in patients with VAH and in addition, a higher venous outflow resistance (due to either jugular valve insufficiency and/or elevated thoracic pressure) resulting in a (temporary) dysfunction of CA-1 neurons, preferably in the vulnerable hippocampal watershed area between the arterial branches form the anterior and the posterior circulation.

Supporting this hypothesis, Piffer et al. proposed that “Acute vascular … events may trigger TGAs … by activating direct pathways within the nervous system leading to TGA, or alternatively elicit a bodily sympathetic overactivity cascade.,” indicating that vascular changes might have an influence not only when resulting in stroke (35).

The most important limitations of our study were that it was a monocentric analysis only and that we used a surrogate parameter instead of assessing hippocampal perfusion directly by dedicated imaging techniques (due to the retrospective design using data from clinical routine only). Its strength is the large number of patients and the comparison to a matched control group.

Further studies should include perfusion weighted imaging of the hippocampus (during the acute stage of TGA) and the assessment of concomitant venous anomalies as these could have a synergistic effect resulting in hippocampal hypoperfusion. The type of hippocampal vascular supply should also be assessed (with high-field MRI) because the single supply type (without inflow of the AChA) might be more vulnerable to reduced vertebral perfusion.

It would also be interesting to perform repeated TOF-MRA imaging (in and after the amnestic episode) because reversible vasoconstriction in the context of TGA has been observed (36).

Potential interactions of VAH with trigger events preceding TGA (regarding type and intensity of the trigger) should also be studied.

## Data availability statement

The data analyzed in this study is subject to the following licenses/restrictions: Original MRA data can be viewed on request. Requests to access these datasets should be directed to mk264@uni-heidelberg.de.

## Ethics statement

The studies involving humans were approved by Ethics committee of the Landesärztekammer Rhineland Palatinate, Mainz, Mittlere Bleiche 40, 55116 Mainz, Germany. The studies were conducted in accordance with the local legislation and institutional requirements. The ethics committee/institutional review board waived the requirement of written informed consent for participation from the participants or the participants' legal guardians/next of kin because the retrospective analysis of the anonymized data (that had been acquired exclusively according to our routine treatment protocol) was in accordance with the directive of the local ethics commission (Rhineland-Palatinate Medical Council) no separate formal approval was required.

## Author contributions

RW: Conceptualization, Data curation, Formal analysis, Investigation, Methodology, Project administration, Validation, Writing – original draft, Writing – review & editing. AE: Formal analysis, Investigation, Validation, Writing – review & editing. IK: Data curation, Formal analysis, Methodology, Validation, Writing – review & editing. JW: Formal analysis, Project administration, Supervision, Validation, Writing – review & editing.
